# Penfluroidol Attenuates the Imbalance of the Inflammatory Response by Repressing the Activation of the NLRP3 Inflammasome and Reduces Oxidative Stress via the Nrf2/HO-1 Signaling Pathway in LPS-Induced Macrophages

**DOI:** 10.1155/2023/9940858

**Published:** 2023-08-22

**Authors:** Qiulin Li, Lidong Wu, Bin Cheng, Shaoyu Tao, Wei Wang, Zhiqiang Luo, Jun Fan

**Affiliations:** Department of Emergency, Second Affiliated Hospital of Nanchang University, Nanchang 330006, China

## Abstract

**Objectives:**

Excessive inflammatory responses and reactive oxygen species (ROS) formation play pivotal roles in the pathogenesis of sepsis. Penfluroidol (PF), an oral long-acting antipsychotic drug, has been suggested to possess diverse biological properties, including antischizophrenia, antitumour effect, and anti-inflammatory activity. The purpose of this research was to explore the anti-inflammatory and antioxidative effects of penfluroidol on lipopolysaccharide (LPS)-related macrophages.

**Methods:**

The viability of RAW264.7 and THP-1 cells was measured by Enhanced Cell Counting Kit-8 (CCK-8). The production of nitric oxide was evaluated by the Nitric Oxide Assay Kit. The generation of pro-inflammatory monocytes was detected by qRT-PCR (quantitative real-time PCR) and ELISA (enzyme-linked immunosorbent assay). Oxidative stress was assessed by measuring ROS, malondialdehyde (MDA), and superoxide dismutase (SOD) activity. The protein expression of the Nrf2/HO-1/NLRP3 inflammasome was detected by western blotting.

**Results:**

Our results indicated that no cytotoxic effect was observed when RAW264.7 and THP-1 cells were exposed to PF (0–1 *μ*m) and/or LPS (1 *μ*g/ml) for 24 hr. The data showed that LPS, which was repressed by PF, facilitated the generation of the pro-inflammatory molecules TNF-*α* and IL-6. In addition, LPS contributed to increased production of intracellular ROS compared with the control group, whereas the administration of PF effectively reduced LPS-related levels of ROS. Moreover, LPS induced the generation of MDA and suppressed the activities of SOD. However, PF treatment strongly decreased LPS-induced MDA levels and increased SOD activities in the RAW264.7 and THP-1 cells. Furthermore, our research confirmed that penfluroidol repressed the secretion of pro-inflammatory molecules by limiting the activation of the NLRP3 inflammasome and reducing oxidative effects via the Nrf2/HO-1 signaling pathway.

**Conclusion:**

Penfluroidol attenuated the imbalance of the inflammatory response by suppressing the activation of the NLRP3 inflammasome and reduced oxidative stress via the Nrf2/HO-1 signaling pathway in LPS-induced macrophages.

## 1. Introduction

Sepsis is a multiorgan dysfunction syndrome characterized by immune dysfunction resulting from infection [1]. Sepsis is associated with a high-morbidity rate and is estimated to affect at least 31.5 million people worldwide each year [2]. The pathogenesis of sepsis is extremely complex; it involves a series of basic problems, such as infection, inflammation, immunity, coagulation, neuroendocrine, and tissue damage, and is closely related to the pathophysiological changes in multiple systems and organs of the body [3, 4]. Therefore, clinical treatment of sepsis is difficult, and sepsis is one of the leading causes of death in critically ill patients [5]. Although the pathogenesis mechanism of sepsis is not completely defined, accumulating clinical and experimental data demonstrate that an excessive inflammatory response and reactive oxygen formation play an important role in the pathogenesis of sepsis [6–9].

The imbalance of the inflammatory reaction in sepsis is manifested in the coexistence of high and low responses [10]. In the initial stage of sepsis, it manifests as a storm of inflammatory factors, which may lead to severe organ damage and death in some patients [11]. The initial high-inflammatory reaction is accompanied by almost simultaneous generation of anti-inflammatory molecules, which leads to immune paralysis in the late stage, in which patients are less likely get rid of the pathogen [12]. In addition, previous studies have provided evidence suggesting that activation of the NLRP3 inflammasome is an important mechanism for the occurrence of an excessive inflammatory response [13]. The NLRP3 inflammasome, the main polyprotein complex, is composed of NLRP3, caspase-1, and apoptosis-related speck-like protein adapter (ASC) [14]. It regulates the activation of caspase-1 and subsequently facilitates the generation of pro-inflammatory monocyte IL-1*β* and IL-18 [15]. On the other hand, reactive oxygen species (ROS) formation is of great importance to enhance the development of sepsis [9]. In the occurrence and development of sepsis, inflammation activated by pathogens and their toxins affects the coupling process of the oxidative respiratory chain in mitochondria, participating in the generation of ROS [16]. ROS can cause lipid oxidation in local tissue structures and destroy cell structures [17]. Importantly, nuclear factor erythroid 2-related Factor 2 (Nrf2) mainly contributes to inhibiting the production of ROS [18]. Upon activation, Nrf2 can regulate heme oxygenase 1 (HO⁃1) to eliminate oxidative stress [19]. As a result, the activation of Nrf2 contributes to protecting RAW264.7 cells against the oxidative damage. Consequently, many studies indicate that repressing excessive inflammation and oxidative stress may be helpful for preventing the mortality in patients with sepsis [6, 9].

Penflulidol, an oral long-acting antipsychotic drug, was synthesized in 1963 [20]. It is mainly used to treat acute psychosis, schizophrenia, and multiple tics by blocking dopamine receptors, especially postsynaptic D2 receptors [21]. Currently, a great deal of research has indicated that PF not only has an antischizophrenia effect but also has strong pharmacological activities, such as anti-inflammatory, antitumor, and antibacterial effects [22–25]. However, to our knowledge, the effect of penfluroidol in sepsis remains largely unknown. LPS-stimulated macrophages are known to be the most common model of inflammation. In this research, we explored the anti-inflammatory and antioxidative effects of penfluroidol on LPS-induced macrophages and relevant molecular mechanisms.

## 2. Materials and Methods

### 2.1. Chemicals and Reagents

Penfluridol (purity ≥ 98.92%) was obtained from MedChemExpress (HY-B1077, Shanghai, China). Lipopolysaccharides (LPS from *Escherichia coli* 055: B5) was acquired from Sigma Chemical Co. (St. Louis, MO, USA). High-glucose DMEM and FBS were obtained from Solarbio (Beijing, China). Enhanced Cell counting Kit-8 (CCK-8) was purchased from Bioss (Beijing, China). Mouse and human ELISA kits for TNF-*α*, IL-6, IL-18, and IL-1*β* were acquired from BOSTER Biological Technology Co. Ltd., (Wuhan, China). The nitric oxide (NO), malondialdehyde (MDA), and superoxide dismutase (SOD) kits were obtained from Beyotime Institute of Biotechnology (Beijing, China). Primary antibodies against NLRP3, cleaved Caspase-1 and Nrf2 were purchased from Cell signaling. Antibodies against HO-1, lamin B, GAPDH, and *β*-tublin were purchased from proteintech Group, Inc (Wuhan, China). Primary antibody against IL-1*β* was purchased from Abcam (ab234437; Abcam, Cambridge, UK). HRP-Goat Anti-Mouse IgG (H + L) and HRP-Goat Anti-Rabbit IgG (H + L) were purchased from proteintech group, Inc (Wuhan, China).

### 2.2. Cell Cultures and Grouping

The mouse macrophage cell lines RAW264.7 and THP-1 were acquired from Procell (Wuhan, China) and cultured in high-glucose DMEM containing 10% foetal bovine serum (FBS), 100 U/ml penicillin and 100 *μ*g/ml streptomycin. RAW264.7 and THP-1 cells were incubated at 37°C in a 5% CO_2_ atmosphere. We divided the RAW264.7 and THP-1 cells into four experimental groups: control group, LPS group, LPS + 0.5 *μ*m PF, and LPS + 1 *μ*m PF.

### 2.3. Cell Viability Assay

RAW264.7 and THP-1 macrophages were plated in 96-well plates at a density of 1 × 10^4^ per well, and exposed to 1 *μ*g/ml LPS and/or different concentrations of penfluridol (0.5, and 1 *μ*m) for 24 hr. Then, we added 10 *μ*l of CCK-8 solution to each well and incubated the plate for 2 hr at room temperature. The absorbance at 420 nm was measured by the Fluoroskan Ascent FL (ThermoFisher, USA).

### 2.4. Measurement of Nitrite (NaNO2) Production

Standard substances and samples were added to a 96-well plate at 50 *μ*l/well. Then, Griess Reagent I and Griess Reagent II were added to each well at 50 *μ*l/well and incubated for 10 min at room temperature. The absorbance at 540 nm was measured by a Fluoroskan Ascent FL (Thermo Fisher, USA).

### 2.5. ELISA for TNF-*α*, IL-6, IL-1*β*, and IL-18

We assessed the levels of TNF-*α*, IL-6, IL-18, and IL-1*β* in the supernatant by mouse and human ELISA kits. The odd ratio (OD) value at 450 nm was determined by Fluoroskan Ascent FL (Thermo Fisher, USA).

### 2.6. Protein Extraction and Western Blot Analysis

Total cell proteins were extracted by RIPA tissue/cell lysate (Solarbio, China). We used a BCA Protein Assay Kit (CWBIO, China) to assess the total protein concentration. To evaluate the expression of protein, we used 10% SDS‒PAGE to isolate the protein samples and transferred the samples to polyvinylidene fluoride (PVDF) membranes. The membranes were incubated with 5% BSA sealer solution for at least 2 hr. Next, the membranes were incubated with primary antibody for 24 hr and then with secondary antibodies for 1–2 hr. Finally, the membranes were developed using Super ECL Plus (UElandy, China). ImageJ software was used for quantitative determination of each strip.

### 2.7. Nuclear and Cytosolic Extract Preparation

RAW264.7 cells nuclear and cytoplasmic protein were separated according to the specifications of the factory. RAW264.7 cells were whirled for 5 s, and then we placed the cells on ice for 15 min. Subsequently, cytoplasmic extraction Reagent B was added to the samples. Next, the samples were centrifuged at 12,000 × *g* for 5 min, and the supernatant contained the cytoplasmic protein. Nucleus extraction reagent was added to the remaining precipitate, repeating the above steps. Finally, the nuclear protein was extracted from the samples.

### 2.8. Measurement of Intracellular Antioxidant Levels

We detected MDA and SOD content in cells by MDA and SOD Assay Kits (Beyotime, China).

### 2.9. Detection of Intracellular ROS Levels

RAW264.7 cells and THP-1 were cultured in 6-well plate at a density of 1 × 10^4^ per well. After the cells attached to the plate, we removed the cell culture medium. Next, PF diluted with serum-free medium was added to the plate. The cells were incubated at 37° for 24 hr away from light. Then, PF was removed, and 2, 7-dichloro-fluorescein diacetate (DCFH-DA) was added to the plate. Finally, the cells were incubated at 37°C for 30 min in the dark. The fluorescence was observed under the microscope.

### 2.10. RT-qPCR Analysis

Total RNA was extracted using TRIzol (CWBIO, China), and the RNA concentration was tested by a UV spectrophotometer. RNA was reverse-transcribed to cDNA by EasyScript One-Step gDNA Removal and cDNA Synthesis SuperMix 100 (Biotech, China). RT-qPCR was performed using Green® Premix Ex Taq™ II (Tli RNaseH Plus) on a Thermo Fisher. The relative expression of mRNAs was calculated by 2^−*ΔΔ*Ct^. The primers used are shown in Table 1.

### 2.11. Statistical Analysis

All statistical analyses were calculated by SPSS22.0. The data are shown as the mean ± SD, and one-way ANOVA was used to compare the differences. A value of *p* < 0.05 was identified as statistically significant.

## 3. Results

### 3.1. Chemical Structure of Penfluroidol and Cell Viability

**Figure 1:**
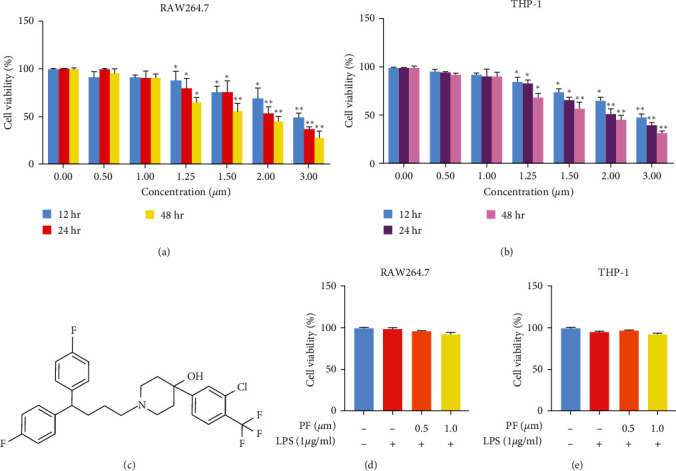
The effect of penfluridol on the viability of macrophages. (a and b) RAW264.7 and THP-1 cells were treated with penfluridol (0, 0.5, 1, 1.25, 1.5, 2, and 3 *μ*m) for 12, 24, and 48 hr, and the effect of penfluridol on the viability of RAW264.7 and THP-1 macrophages was detected by Enhanced Cell Counting Kit-8 (CCK-8). (c) Chemical structure formula of penfluridol. (d) The effect of penfluridol on LPS-induced RAW264.7 cell activity. (e) The effect of penfluridol on LPS-induced THP-1 cell activity. The data are shown as the mean ± SD, and one-way ANOVA was used to compare the differences.  ^*∗*^*p* < 0.05 and  ^*∗*^ ^*∗*^*p* < 0.01.

Penfluridol is a diphenylbutylpiperidine compound. The molecular weight of penfluridol is 523.97 [26]. The chemical structure formula is shown in Figure 1(c). As shown in Figures 1(a) and 1(b), RAW264.7 and THP-1 cells were treated with penfluridol (0, 0.5, 1, 1.25, 1.5, 2, and 3 *μ*m) for 12, 24, and 48 hr. Then, we used an CCK-8 to measure the effect of penfluridol on the viability of RAW264.7 and THP-1 macrophages. No cytotoxic effect was observed when cells were exposed to penfluridol (0–1 *μ*m), but the viability of RAW264.7 and THP-1 macrophages decreased when exposed to a concentration of penfluridol ≥1.25 *μ*m. As shown in Figures 1(d) and 1(e), LPS-stimulated RAW264.7 and THP-1 macrophage activity were not affected by penfluridol. To ensure that the anti-inflammatory and antioxidant activities were not affected by cell viability, we chose noncytotoxic concentrations of penfluridol (0.5 and 1 *μ*m) in the following experiments.

### 3.2. Penfluroidol Alleviates LPS-Induced Inflammation in LPS-Stimulated Macrophages

**Figure 2:**
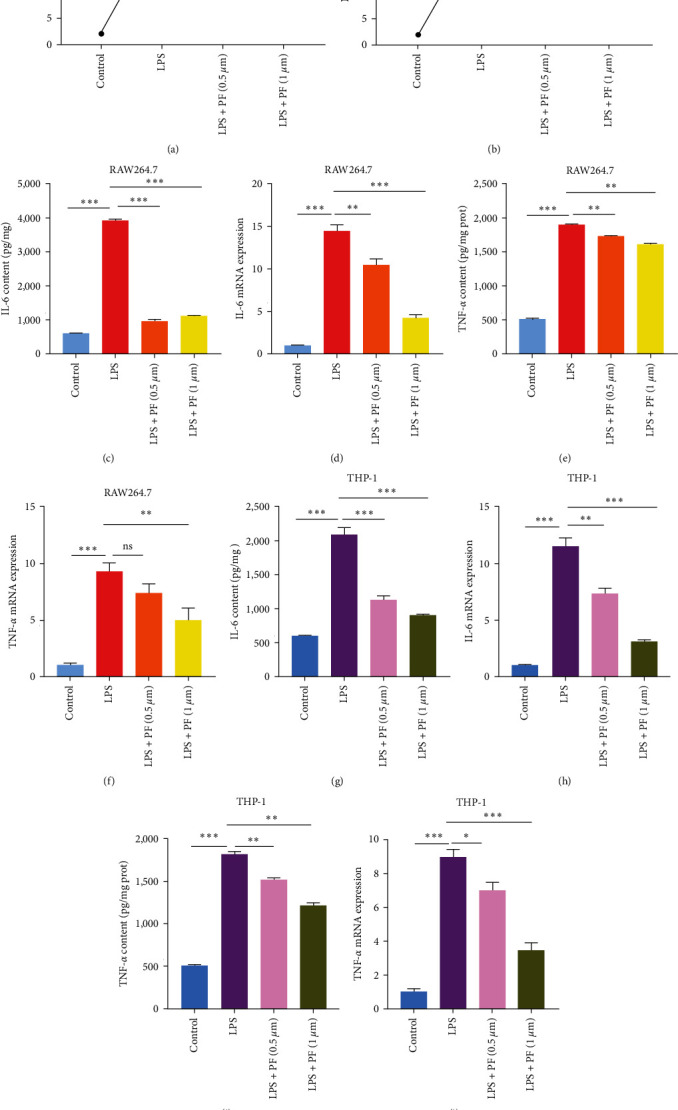
PF alleviated LPS-induced inflammation in LPS-stimulated macrophages. RAW264.7 and THP-1 cells were treated with PF for 24 hr and stimulated with LPS (1 *μ*g/ml) for 24 hr. (a and b) The level of NO was measured by Griess Reagent. (c–f) The concentrations of IL-6 and TNF-*α* were detected by ELISA and qRT-PCR in treated RAW264.7 cells. (g–j) The levels of IL-6 and TNF-*α* were assessed by qRT-PCR and ELISA in treated THP-1 cells. The data are shown as the mean ± SD, and one-way ANOVA was used to compare the differences.  ^*∗*^*p* < 0.05,  ^*∗*^ ^*∗*^*p* < 0.01, and  ^*∗*^ ^*∗*^ ^*∗*^*p* < 0.001.

To evaluate the anti-inflammatory effect of PF on LPS-stimulated RAW264.7 and THP-1 macrophages, we measured the production of NO and assessed the generation of proinflammatory monocyte TNF-*α* and IL-6. As shown in Figures 2(a) and 2(b), LPS promoted the generation of NO compared with the control group. However, PF treatment notably reduced the production of NO compared with the LPS treatment. TNF-*α* and IL-6 are typical proinflammatory markers. As shown in Figure 2(c)–2(j), LPS increased the mRNA expression and secretion of pro-inflammatory monocytes (TNF-*α* and IL-6) compared with that in the control group, which was inhibited by PF. Overall, our study showed that PF diminished the generation of proinflammatory monocytes in LPS-related RAW264.7 and THP-1 cells.

### 3.3. Penfluroidol Inhibits the Activation of the NLRP3 Inflammasome in LPS-Stimulated RAW264.7 Cells

**Figure 3:**
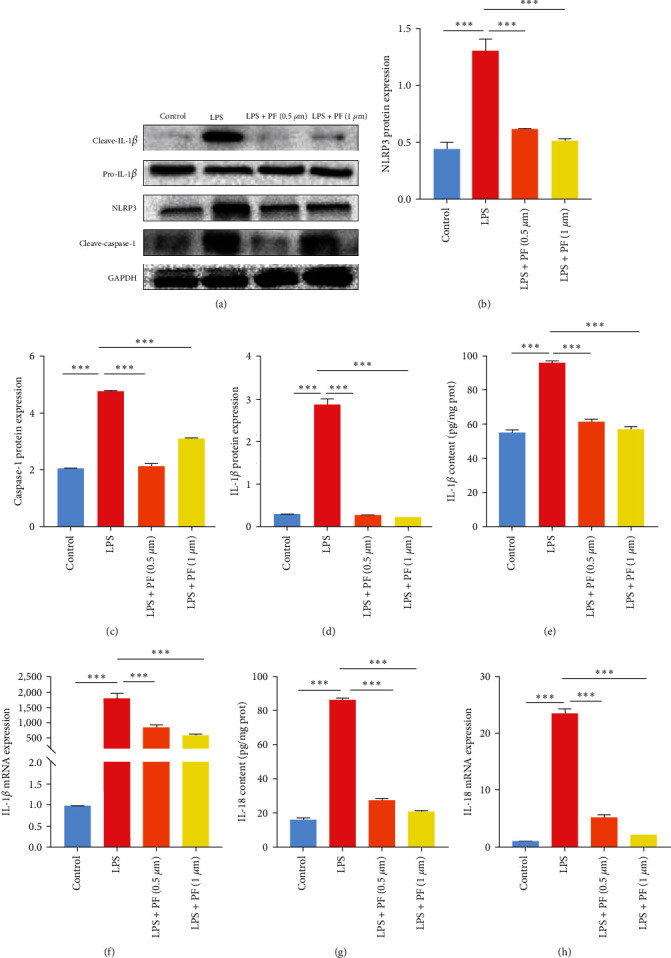
PF inhibited activation of the NLRP3 inflammasome in LPS-stimulated RAW264.7 cells. RAW264.7 cells were treated with PF for 24 hr and stimulated with LPS (1 *μ*g/ml) for 24 hr. (a–d) Western blotting technology was used to investigate the protein expression of NLRP3, cleave-caspase-1, and IL-1*β*. (e and f) The level of IL-1*β* was measured by ELISA and qRT-PCR. (g and h) The content of IL-18 was assessed by ELISA and qRT-PCR. The data are shown as the mean ± SD, and one-way ANOVA was used to compare the differences.  ^*∗*^*p* < 0.05,  ^*∗*^ ^*∗*^*p* < 0.01, and  ^*∗*^ ^*∗*^ ^*∗*^*p* < 0.001.

Furthermore, to investigate the possible anti-inflammatory mechanism of PF, we evaluated the expression of NLRP3, cleaved capcase-1, IL-1*β*, and IL-18. As shown in Figure 3(a)–3(d), LPS significantly facilitated the protein expression of NLRP3 and cleaved capcase-1 and IL-1*β*. However, PF treatment effectively abrogated the protein expression of NLRP3, cleaved caspase-1, and IL-1*β* compared with LPS administration. As shown in Figure 3(e)–3(h), the levels of IL-1*β* and IL-18 were measured by ELISA and qRT-PCR. The results demonstrated that LPS increased the mRNA expression and secretion of pro-inflammatory monocytes (IL-1*β* and IL-18) compared with the control group, which was inhibited by PF. In summary, our data indicated that PF obviously blocked the LPS-related activation of the NLRP3 inflammasome in RAW264.7 cells.

### 3.4. Penfluroidol Reduces Oxidative Stress in LPS-Related Macrophages

**Figure 4:**
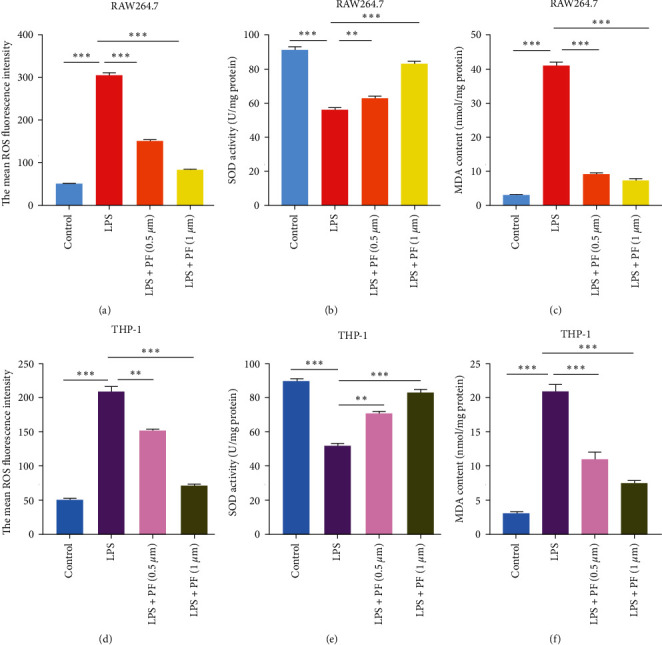
PF reduced oxidative stress in LPS-stimulated macrophages. RAW264.7 and THP-1 cells were treated with PF for 24 hr and stimulated with LPS (1 *μ*g/ml) for 24 hr. (a) The level of ROS was measured by the 2, 7-dichloro-fluorescein diacetate (DCFH-DA) in treated RAW264.7 cells. (b and c) SOD and MDA contents in RAW264.7 cells were detected by MDA and SOD Assay Kits. (d) The level of ROS was measured by the 2, 7-dichloro-fluorescein diacetate (DCFH-DA) in treated THP-1 cells. (e and f) SOD and MDA contents in THP-1 cells were detected by MDA and SOD Assay Kits. The data are shown as the mean ± SD, and one-way ANOVA was used to compare the differences.  ^*∗*^*p* < 0.05 ^*∗*^ ^*∗*^*p* < 0.01, and  ^*∗*^ ^*∗*^ ^*∗*^*p* < 0.001.

To confirm the antioxidant properties of PF in LPS-stimulated macrophages, we measured the generation of intracellular ROS, MDA, and SOD. As shown in Figure 4(a), the fluorescence image results indicated that LPS contributed to the production of intracellular ROS, whereas the administration of PF distinctly reduced LPS-related levels of ROS in RAW264.7 cells. In addition, we also evaluated the antioxidant enzyme activity of SOD and the oxidative stress marker MDA content in RAW264.7 cells by MDA and SOD Assay Kits (Beyotime, China). As shown in Figures 4(b) and 4(c), our results indicated that treatment with LPS induced the production of MDA and suppressed the activities of SOD compared with the control group. However, PF treatment strongly decreased LPS-induced MDA levels and increased SOD activities in RAW264.7 cells. To demonstrate the antioxidant properties of PF, we also measured the generation of intracellular ROS, MDA, and SOD in THP-1 cells. As shown in Figure 4(d), our results indicated that LPS contributed to the level of intracellular ROS, whereas the administration of PF distinctly reduced LPS-related production of ROS in THP-1 cells. As shown in Figures 4(e) and 4(f), treatment with LPS induced the production of MDA and suppressed the activities of SOD compared with the control group. However, PF treatment strongly decreased LPS-induced MDA levels and increased SOD activities in THP-1 cells. Overall, these results clearly indicated that PF protected RAW264.7 and THP-1 cells against the LPS-related oxidative damage.

### 3.5. Penfluroidol Exerts Antioxidant Activity by Promoting the Nuclear Translocation of Nrf2 in LPS-Stimulated RAW264.7 Cells

**Figure 5:**
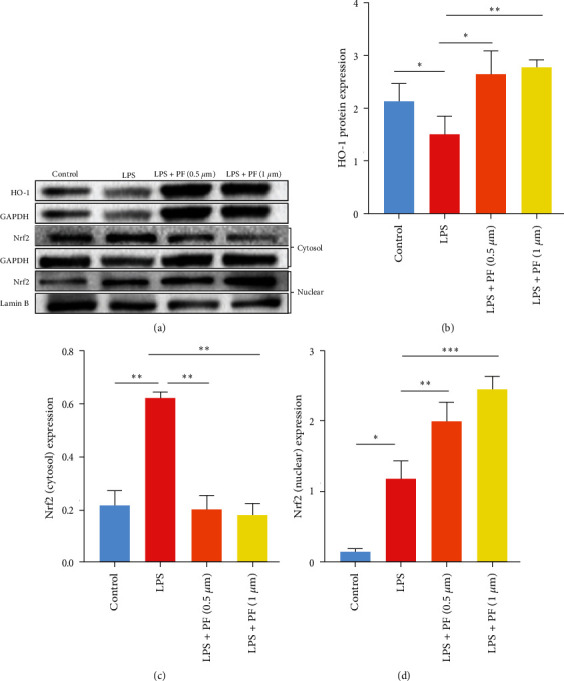
PF promoted the nuclear translocation of Nrf2 and facilitated the expression of HO-1 in LPS-stimulated RAW264.7 cells. RAW264.7 cells were treated with PF for 24 hr and stimulated with LPS (1 *μ*g/ml) for 24 hr. Western blotting technology was used to investigate the protein expression of HO-1, nuclear Nrf2, and cytosolic Nrf2. The data are shown as the mean ± SD, and one-way ANOVA was used to compare the differences  ^*∗*^*p* < 0.05,  ^*∗*^ ^*∗*^*p* < 0.01, and  ^*∗*^ ^*∗*^ ^*∗*^*p* < 0.001.

Furthermore, to explore the antioxidant mechanism of penfluridol, we used western blotting technology to investigate the protein expression of Nrf2 and HO-1. As shown in Figure 5, the administration of PF effectively induced the production of nuclear Nrf2 and repressed the expression of cytosolic Nrf2, and the PF treatment visibly elevated the expression of HO-1 compared with the LPS group. The above data clearly revealed that penfluridol downregulated oxidative stress by promoting the nuclear translocation of Nrf2 protein and upregulating the expression of HO-1. Therefore, we speculated that PF alleviated antioxidant activity by regulating the Nrf2/HO-1 signaling pathway.

## 4. Discussion

### 4.1. The First Meaningful Finding of This Study Was That Penfluroidol Attenuated the Imbalance of the Inflammatory Response by Inhibiting Activation of the NLRP3 Inflammasome in Macrophages

The NLRP3 inflammasome is the main polyprotein complex [14]. Many studies have reported the effect of the NLRP3 inflammasome on the pathogenesis of sepsis [27, 28]. Busch et al. [27] found that the formation of the NLRP3 inflammasome resulted in the cardiomyopathy of polymicrobial sepsis. Shi et al. [28] also found that activation of the NLRP3 inflammasome contributed to endothelial dysfunction by enhancing the expression of tissue factor (TF) in sepsis. Consequently, suppressing the activation of the NLRP3 inflammasome may be good for patients with sepsis. Fu et al. [29] demonstrated that limiting the activation of the NLRP3 inflammasome could attenuate cognitive impairment in septic animals. Moreover, Cornelius et al. [30] found that restraint of the NLRP3 inflammasome alleviates sepsis-related activation of platelets and limits multiorgan dysfunction in cacal ligation puncture. In addition, Lee et al. [31] found that the absence of the NLRP3 inflammasome increased the expression of lipoxin B4 synthesized against the microbial sepsis. Moreover, many studies have found that inhibiting the activation of the NLRP3 inflammasome reduces sepsis-induced acute lung damage [32] and attenuates sepsis-induced acute kidney injury [32], and attenuates sepsis-induced acute kidney injury [33]. In this study, our data confirmed that LPS obviously promoted the secretion of proinflammatory monocytes in RAW264.7 and THP-1 macrophages. However, PF treatment distinctly decreased LPS-related levels of proinflammatory cytokines. These data showed that PF treatment repressed the excessive inflammatory response in macrophages. Chen et al. [22] found that PF prevented the generation of inflammatory monocytes by binding to acid sphingomyelinase in arthritis and colitis. Furthermore, to explore the potential anti-inflammatory mechanism of PF, we evaluated the expression of NLRP3, cleaved caspase-1, IL-1*β*, and IL-18. As shown in Figure 3(a)–3(d), LPS significantly facilitated the protein expression of NLRP3, cleaved caspase-1, and IL-1*β*. However, PF treatment effectively abrogated the protein expression of NLRP3, cleaved caspase-1, and IL-1*β* compared with LPS administration. As shown in Figure 3(e)–3(h), the levels of IL-1*β* and IL-18 were measured by ELISA and qRT-PCR. The results demonstrated that LPS increased the mRNA expression and secretion of proinflammatory monocytes (IL-1*β* and IL-18) compared with the control group, which was inhibited by PF. In summary, our data indicated that PF obviously blocked the LPS-related activation of the NLRP3 inflammasome in RAW264.7 cells. Therefore, our data showed that PF reduced the release of pro-inflammatory monocytes by limiting the activation of the NLRP3 inflammasome, thereby alleviating the imbalance in the inflammatory response in patients with sepsis.

### 4.2. The Second Most Valuable Finding of This Study Was That Penfluroidol -Reduced Oxidative Stress via the Nrf2/HO-1 Signaling Pathway in LPS-Induced RAW264.7 Macrophages

Oxidative stress is related to the imbalance between the generation of ROS and the consumption of antioxidant enzymes [34]. Oxidative stress can result in a series of problems in sepsis [35–37]. For examples, oxidative stress can cause mitochondrial injury, which is fundamental to the pathophysiology of organ failure in sepsis [35]. Moreover, oxidative damage initiates the inflammatory response by activating the NF-*k*B signaling pathway [36]. In addition, oxidative stress has a negative effect on organ dysfunction in animal models [37]. Hence, inhibiting oxidative stress may be beneficial to patients with sepsis. Liu et al. [38] confirmed that repressing oxidative effects alleviated sepsis-related acute lung injury. Mei et al. [39] found that suppressing oxidative effects reduced cerebral ischemia injury in vivo and in vitro. Peng et al. [40] demonstrated that restraining oxidative stress diminished hypoxia-induced inflammation in BV2 microglia. Accumulating studies have indicated that the main cause of oxidative damage is the generation of ROS and the consumption of antioxidant enzymes [34]. To investigate the antioxidant effect of PF in LPS-related macrophages, we established a model of LPS-stimulated RAW264.7 and THP-1 macrophages. We measured the level of ROS by 2,7-dichloro-fluorescein diacetate (DCFH-DA) and assessed the production of MDA and SOD by MDA and SOD Assay Kits. Our data showed that LPS led to the generation of intracellular ROS in LPS-induced RAW264.7 and THP-1 macrophages. However, PF treatment effectively reduced LPS-related levels of ROS. Similarly, LPS induced the generation of MDA and suppressed the activities of SOD in RAW264.7 and THP-1 macrophages. However, treatment with PF strongly decreased the activities of MDA and enhanced the activities of the antioxidant enzyme SOD. Our research indicated that PF protected RAW264.7 and THP-1 macrophages against oxidative stress. Furthermore, to probe the antioxidant mechanism of PF, we explored the effect of PF on the expression of key proteins in the oxidative stress signaling pathway. The data showed that PF increased the protein expression of nuclear Nrf2 in LPS-induced macrophages. Many studies have confirmed that the major function of Nrf2 is resistance to oxidative stress [41, 42]. Audi et al. [41] verified that compared with WT rats, Nrf2 knockout rats suffered from greater hyperoxia-stimulated histological damage. Qi et al. [42] confirmed that activation of Nrf2 could protect HepG2 cells against oxidative damage. Specifically, upon activation, Nrf2 translocates to the nucleus and combines with the antioxidant response element, which regulates the expression of HO-1 [19]. HO-1, an antioxidant enzyme, can suppress oxidative effects by regulating the generation of excessive ROS [43]. Our results suggested that PF exerted an antioxidant role by facilitating the nuclear translocation of Nrf2 and thereby upregulating the expression of HO-1.

## 5. Conclusion

**Figure 6:**
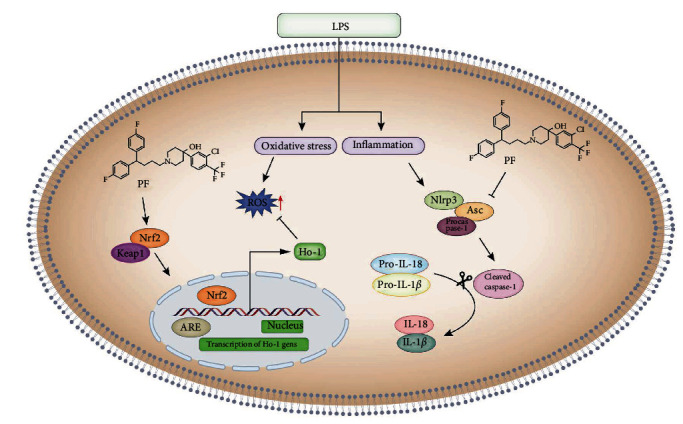
Graphical representation for penfluroidol treatment attenuates the imbalance of the inflammatory response and reduces oxidative stress.

In summary, the study indicated that PF blocked the secretion of pro-inflammatory monocytes by suppressing the activation of the NLRP3 inflammasome and repressed oxidative stress via the Nrf2/HO-1 signaling pathway in LPS-related macrophages (Figure 6). Clinically, regulating the imbalance of inflammation and reducing the oxidative stress may have a beneficial effect on the prognosis of patients with sepsis. However, due to the limitations of our current research, we do not explain in detail how PF affects the Nrf2/HO-1/NLRP3 inflammasome signal pathway. Therefore, we need to study the specific mechanism in the future.

## Figures and Tables

**Table 1:** The primer sequences in the current study.

Gene	Forward primers (5′-3′)	Reverse primers (5′-3′)
GAPDH (mouse)	TGGAAAGCTGTGGCGTGATG	TACTTGGCAGGTTTCTCCAGG
TNF-*α* (mouse)	GGACTAGCCAGGAGGGAGAACAG	GCCAGTGAGTGAAAGGGACAGAAC
IL-6 (mouse)	CTTCTTGGGACTGATGCTGGTGAC	TCTGTTGGGAGTGGTATCCTCTGTG
IL-1*β* (mouse)	CACTACAGGCTCCGAGATGAACAAC	TGTCGTTGCTTGGTTCTCCTTGTAC
IL-18 (mouse)	ACCTGAAGAAAATGGAGACCTGG	AGTCATATCCTCGAACACAGGC
GAPDH (homo)	GGTGTGAACCATGAGAAGTATGA	GAGTCCTTCCACGATACCAAAG
TNF-*α* (homo)	TCCTCTCTGCCATCAAGAGC	AGTAGACCTGCCCAGACTCG
IL-6 (homo)	ATGCAATAACCACCCCTGAC	GCGCAGAATGAGATGAGTTGT
IL-1*β* (homo)	GCTTATTACAGTGGCAATGAGGAT	TAGTGGTGGTCGGAGATTCG
IL-18 (homo)	GACCAAGGAAATCGGCCTCTA	ACCTCTAGGCTGGCTATCTT

## Data Availability

The original data used to support the findings of this study are available from the corresponding author upon request.
